# Multi-omics analysis revealed the differences in lipid metabolism of the gut between adult and juvenile yellowfin tuna (*Thunnus albacares*)

**DOI:** 10.3389/fmicb.2023.1326247

**Published:** 2024-01-11

**Authors:** Ying Zou, Yanjie Zhang, Di Wu, Zhiyuan Lu, Juan Xiao, Hai Huang, Qiongyao Fu, Zhiqiang Guo

**Affiliations:** ^1^School of Life and Health Sciences, School of Marine Science and Engineering, School of Food Science and Engineering, State Key Laboratory of Marine Resource Utilization in South China Sea, Hainan University, Haikou, China; ^2^Key Laboratory of Utilization and Conservation for Tropical Marine Bioresources, Hainan Key Laboratory for Conservation and Utilization of Tropical Marine Fishery Resources, College of Fisheries and Life Science, Hainan Tropical Ocean University, Sanya, China; ^3^Key Laboratory of Tropical Translational Medicine of Ministry of Education, NHC Key Laboratory of Control of Tropical Diseases, School of Tropical Medicine, Hainan Medical University, Haikou, Hainan, China

**Keywords:** *Thunnus albacares*, gut microbiota, 16S rRNA sequencing, metabolome, transcriptome

## Abstract

**Introduction:**

Tuna has a cost-effective energy supply to support the regional endothermic and high-speed swimming performance. The gut symbiotic microbiotas and their metabolites play essential roles in tuna’s diet digestion, absorption, and energy acquirement, which are often highly related to the ontogenetic development of tuna.

**Methods:**

We compared gut microbial compositions and metabolites, as well as mRNA expression of the intestine between juvenile and adult yellowfin tuna using 16S rRNA sequencing, metabolomic and transcriptomic, respectively.

**Results and discussion:**

The results revealed that adults had a significantly higher microbial diversity and abundance of *Acinetobacter* than juveniles. Regarding the gut microbiota-derived metabolites, fatty acids, especially glycerophospholipid and sphingolipid, were significantly enriched in adults than in juveniles. Moreover, the short-chain fatty acid (butyrate and isobutyrate) contents were significantly higher in adults than in juveniles. To find the relationship between gut microbiotas and host physiology, intestinal transcriptome analysis demonstrated that the enriched pathways of differential expression genes (DEGs) in adult tuna were the lipid metabolism pathway, including “fat digestion and absorption,” “cholesterol metabolism,” “steroid hormone biosynthesis,” “glycerolipid metabolism,” and “glycerophospholipid metabolism.” However, protein digestion and absorption and pancreatic secretion pathways were significantly enriched in the juveniles. The conjoint analysis indicated that the enriched pathways of both differential metabolites (DMs) and DEGs were remarkably related to the regulation of glycerophospholipids metabolism in adult tunas. This study highlights the role of gut microbiotas in fish nutrition metabolism. These findings provide new insights into the view of ontogenetic shifts of gut microbiotas and their metabolites on host health and gut function in endothermic and high-speed swimming marine fish species.

## 1 Introduction

Tuna is considered unique in physiology because of their regional endothermy (Block et al., 1993, 2001). They can keep the internal peritoneal temperature stable at approximately 25°C to 28°C and approximately 10°C temperature difference from the body surface (Block et al., 2001). The thermogenic source of tuna is attributed to contractions of the slow-twitch aerobic red muscles during constant high-speed swimming (Block et al., 1993). Heat production is beneficial to deep-sea predation for tuna, but it costs a lot of energy. Intestinal energy harvest from dietary nutrients is an important initial step for the complex physiologic network determining energy balance (Wen and Rawls, 2020). It has been evidenced that gut microbiotas and its metabolites are intimately related to energy absorption and utilization (Rowland et al., 2018; Butt and Volkoff, 2019). Moreover, the ontogenetic dietary shift is widespread in fish (Sánchez-Hernández et al., 2019), which would affect gut microbial composition and diversity (Parata et al., 2020; Ou et al., 2021). However, the characteristics and function of gut microbiota in endothermic tuna are less known.

The gut microbiome plays a crucial role in many physiological processes such as digestion and nutrient absorption, immunoregulation, and epithelial proliferation in animals (Gilbert et al., 2018; Butt and Volkoff, 2019). Firmicutes can stimulate fatty acid uptake and lipid droplet formation in the intestinal epithelium and liver of zebrafish (Semova et al., 2012). *Bacteroides*, *Clostridium*, *Propionibacterium*, *Fusobacterium*, and *Lactobacillus* are evidenced to play an important role in the process of protein hydrolysis (Zhao et al., 2018). Furthermore, gut microbiotas can potentially regulate the expression of approximately 10% of host genes, thereby regulating host epigenetic mechanisms (Camp et al., 2014; Sommer et al., 2015). Sheng et al. (2018) revealed that microbiotas in zebrafish increased lipid accumulation of intestinal epithelium and mRNA expression related to lipid metabolism.

The cross-talk between gut microbiotas and hosts is largely attributed to microbiota-derived metabolites, which are important components in shaping the expression profile of metabolic enzymes of the host and thus modulating nutrient harvest and utilization (Krautkramer et al., 2021; Li et al., 2022; Liu et al., 2022). Microbiota-derived metabolites are also critical signals to intestinal enterocytes regulating transcriptional activity (Hubbard et al., 2015; Martinez-Guryn et al., 2018; Pan et al., 2018; Cani et al., 2019). Butyrate, for instance, can activate the expression of *mucin2*, which promotes intestinal epithelial integrity (Burger-van Paassen et al., 2009). *Clostridium bifermentans* and its bioactive metabolites could selectively induce the expression of critical esterification enzymes and upregulate lipid absorption genes in mouse intestinal epithelium (Martinez-Guryn et al., 2018). Foley et al. (2022) shed light on the expression of P-glycoprotein, a key component of the intestinal epithelium, which is directly regulated by intestinal microbial metabolites, secondary bile acids, and short-chain fatty acids (SCFAs). Zhang et al. (2020) revealed that dietary acetate could promote the appetite and food intake of zebrafish through neuroendocrine regulation, participated by the ghrelin and neuropeptide Y in the brain.

Microbiota-derived metabolites also communicate with other gut bacteria, and multiple interacted communities may have additive, synergistic, redundant, or even subtractive effects on the host (Liu et al., 2022). For example, gut microbiota metabolites, SCFAs, can serve as energy sources for other gut microbiotas, and a high concentration of SCFAs can regulate the lumen pH and inhibit the growth of some gut bacteria, and thus indirectly regulate the composition and function of gut microbial member (Sun and O’Riordan, 2013). However, there is limited information on how the gut microbiota and its metabolites contribute to tuna’s physiology, particularly in nutrient metabolite, or how the microbial members change with host life history (e.g., juvenile vs. adult).

In this study, therefore, the gut microbiota and microbiota-derived metabolite profiles of wild yellowfin tunas (*Thunnus albacares*) were investigated with a multi-omics approach. The goals of this study were to determine (1) whether gut microbial structure was different among juvenile and adult tunas, (2) the differences of gut microbial metabolites among juvenile and adult tunas, and (3) the influence of microbiota-derived metabolites on intestinal gene expression of host. This study aimed to provide a better understanding of the relationship between the symbiotic microbes and host nutrient digestion and absorption in endothermic and high-speed swimming marine fish species.

## 2 Materials and methods

### 2.1 Sample collection

Wild yellowfin tunas were captured by line lures from the South China Sea (17°24′N, 110°36′E) in August 2021. A total of 8 juvenile (J) and 8 adult (A) yellowfin tunas were divided by fork length and body weight (Supplementary Table S1). Tricaine methanesulfonate (MS-222) was used for fish anesthesia and 75% ethanol was used to disinfect the body surface of tunas. The whole intestinal tract was dissected and divided into three sections: foregut, midgut, and hindgut. Gut contents of the foregut were squeezed out by sterilized scissors and tweezers to analyze the microbiotas of gut contents (C) and metabolites. The mucosa layer was scraped by the blade after being washed three times with 1 × PBS buffer to analyze microbiotas of gut mucosal (M). The remanent foregut tissue was collected to analyze the mRNA expression level and enzyme activity. All the biological samples were saved in sterilized tubes, quickly frozen in liquid nitrogen, and transferred to a − 80°C refrigerator until the next procedure.

### 2.2 Microbial 16S amplicon sequencing and data analysis

The extraction of total DNA and construction of 16S rRNA V3-V4 hypervariable regions libraries were done using a FastDNA® Spin Kit for Soil (MP Biomedicals, USA) and NEXTflex^TM^ Rapid DNA-Seq Kit (Bioo Scientific, USA), respectively. All raw reads sequenced by the Illumina MiSeq PE300 platform were quality-controlled, merged, and trimmed by fastp version 0.20.0 (Chen et al., 2018). Operational taxonomic units (OTUs) were clustered with a 97% similarity threshold in Uparse version 7.0.1090 (Edgar, 2013). The sequence taxonomy was identified by the RDP Classifier Bayesian algorithm against the Silva 16S rRNA database (version 1.3.8), with a default confidence threshold of 0.7. All raw reads were deposited in the NCBI Sequence Read Archive (SRA) database (BioProject ID: PRJNA1018001).

Alpha diversity indices (Sobs index, Simpson index, Shannoneven index, and Pd index) of the microbiome were estimated using Mothur (v1.30.2). Significant differences in alpha diversity indices were tested by Welch’s t-test at the OTU level. Beta diversity analysis results were visualized by the principal coordinates analysis (PCoA) clustered at the OTU level based on Bray-Curtis metrics. Species differences between adult and juvenile groups were analyzed by the Wilcoxon rank-sum test.

### 2.3 Metabolites extraction and LC–MS analysis

Six fish in each group were prepared to extract metabolites of gut contents. The 50 mg of gut contents were mixed with a quadruple volume mixture of 400 μL methanol–water (4:1) solution with 0.02 mg/mL L-2-chlorophenylalanin as an internal standard, then sonicated at 40 kHz for 30 min at 5°C, and centrifugated at 13000 × g at 4°C for 15 min. Extracted metabolites were centrifugated for 15 min at 13000 × g at 4°C and the supernatant was retained for the next analysis. The quality control (QC) sample was prepared by mixing each isovolumetric sample and injected at regular intervals (every 10 samples) to monitor the stability of the analysis.

The 2 μL of samples were performed by an ultra-performance liquid chromatography (UPLC) system (AB Sciex, Framingham, US) with an HSS T3 column (100 mm × 2.1 mm, 1.8 μm). Solvent A is 0.1% formic acid in water–acetonitrile (95:5), and solvent B is 0.1% formic acid in acetonitrile–isopropanol–water (47.5, 47.5: 5). The solvent gradient changed according to the following conditions: 0% B—24.5% B over 0–3.5 min; 24.5% B—65% B over 3.5–5 min; 65% B—100% B over 5–5.5 min; 100% B—100% B over 5.5–7.4 min; 100% B—51.5% B over 7.4–7.6 min; 51.5% B—0% B from 7.6 to 7.8 min; and 7.8–10 min holding at 0% B at a flow rate of 0.40 mL/min, with temperature at 40°C. The mass spectrometric data were collected using a UHPLC-Q Exactive HF-X Mass Spectrometer (Thermo Fisher Scientific, MA, United States) equipped with an electrospray ionization (ESI). The MS was operated separately in both positive and negative ion modes. The abovementioned steps were conducted by Majorbio Bio-Pharm Technology Co., Ltd. (Shanghai, China).

The raw data processing was carried out by Progenesis QI (Waters Corporation, Milford, United States) software. Identification of metabolites was matched with online HMDB^1^ and METLIN^2^ databases and a self-built library. Multivariate analysis was performed using the partial least squares discriminant analysis (PLS-DA) using the R package “ropls” to understand global metabolic differences between the adult and juvenile groups. The differential metabolites (DMs) between the pairwise comparison groups were determined by variable important for the projection (VIP) values (VIP > 1) combined with “*value of p* <0.05 and foldchange >1.” The enrichment of DMs was performed in the Kyoto Encyclopedia of Genes and Genomes (KEGG) database.

### 2.4 SCFAs preparing and quantifying

The content of SCFAs in gut content was quantitatively analyzed by LC-ESI-MS/MS. The 50 μL of each sample was homogenized in 100 μL acetonitrile, cryogenically sonicating for 30 min (40 KHz) at 5°C and centrifuging at 13000 × g for 15 min at 4°C. The supernatant reacted with isopycnic 3-nitrophenylhydrazine hydrochloride (200 mM) and 1-ethyl-3-(3-dimethylaminopropyl) carbodiimide hydrochloride (120 mM, containing 6% pyridine) solution at 40°C for 30 min, and then diluted with 50% acetonitrile for detection. Metabolites were separated using an ExionLC^™^ AD system (AB Sciex, United States) equipped with a BEH C18 (150*2.1 mm, 1.7 μm; Waters, United States), followed by UHPLC coupled with QTRAP^®^ 6,500+ mass spectrometer (AB Sciex, United States) equipped with the electrospray ionization (ESI) source at the negative mode.

The results were presented as means ± standard error means (SEM) and visualized by GraphPad Prism 9. Statistics were performed by the Student’s t-test by SPSS version 26.0.

### 2.5 Intestinal enzyme activity analysis

The gut tissue (*n* = 4) was homogenized in sterilized physiological saline (0.86%, pH = 7.4; 1:9, w/v) by a handheld homogenizer, then centrifuged for 20 min (3,000 r/min, 4°C). The supernatant was used for enzyme activity analysis of trypsin, lipase, and α-amylase using commercial kits (Nanjing Jiancheng Bioengineering Institute, China). The data were presented as means ± standard error means (SEM) and visualized by GraphPad Prism 9. Statistics were performed by the Mann–Whitney U test.

### 2.6 Transcriptome analysis and RT-qPCR verifying

Total RNA was extracted from gut tissue samples by TRIzol^®^ Reagent (Invitrogen, CA, United States) according to the manufacturer’s instructions. RNA quantity and integrity were determined using the NanoDrop 2000 (Thermo Fisher Scientific, MA, United States) and Bioanalyzer 2,100 (Agilent Technologies, Germany), respectively. The RNA-seq transcriptome libraries were constructed from purified poly (A) mRNA by TruSeq^™^ RNA sample preparation kit (Illumina, CA, United States) and then synthesized double-stranded cDNA using a SuperScript double-stranded cDNA synthesis kit (Invitrogen, CA, United States) with random hexamer primers. A-tailing and adapter were added to complete terminal repair. The final libraries were sequenced on the Illumina NovaSeq 6,000 platform (Illumina, United States) to produce 2 × 150 bp paired-end reads in Shanghai Majorbio Bio-pharm Biotechnology Co., Ltd. (Shanghai, China). The transcriptomic raw data were deposited in the SRA database (BioProject ID: PRJNA1017392). The raw data were trimmed and quality-controlled by SeqPrep^3^ and Sickle^4^ with default parameters. Then clean reads were separately mapped to the genome of *Thunnus albacares* with orientation mode using HISAT2^5^ software. The mapped reads of each sample were assembled by StringTie^6^ in a reference-based approach.

The count matrix for differential expression genes (DEGs) analysis was generated by RSEM. DEGs between juvenile and adult groups were determined using the DESeq2, with *p*-adjust value ≤0.05 and |log_2_(Fold Change)| > 1. Gene functions were annotated by the gene ontology (GO) and KEGG. GO and KEGG functional enrichment analyses of DEGs were carried out by Goatools and KOBAS, with a *p*-adjust value <0.05. To assess the function and enriched KEGG pathway of all genes, gene set enrichment analysis (GSEA) was performed with GSEA software.^7^ RT-qPCR was carried out to verify the expression pattern of DEGs using the LightCycler 480 Real-Time PCR System (Roche, United States). β-actin was used as the reference gene. The RT-qPCR reaction system was prepared by 2 × ChamQ Universal SYBR qPCR Master Mix (Vazyme Biotech, China). The information on primers and amplification systems is shown in Supplementary Table S2. The relative mRNA expression levels of the selected genes were calculated using the 2^-ΔΔCt^ method.

### 2.7 Multi-omics comprehensive analyses

To assess the association between bacteria and metabolites, correlation analysis were performed between the top 20 abundant genera of mucosal microbiotas and the top 40 abundant metabolites. The correlation was calculated by Pearson correlation coefficient and visualized as heatmap plots using the pheatmap package in R (version 3.3.1). To assess the common KEGG pathways participated by DMs and DEGs, a Venn plot was used to display the number of common pathways annotated by DMs and DEGs, and common KEGG enrichment pathways were shown as barplot, visualized by GraphPad Prism 9.

## 3 Results

### 3.1 Microbial diversity and composition

In this study, 16 microbial samples from gut contents and 14 samples from mucus of tunas were sequenced successfully. A total of 1,581,022 sequences were subsampled and 6,736 OTUs were obtained, representing 56 phyla, 167 classes, 395 orders, 690 families, and 1,603 genera. The number of sequences per sample ranged from 30,180 to 87,614, with an average length of 420 bp.

**Figure 1:**
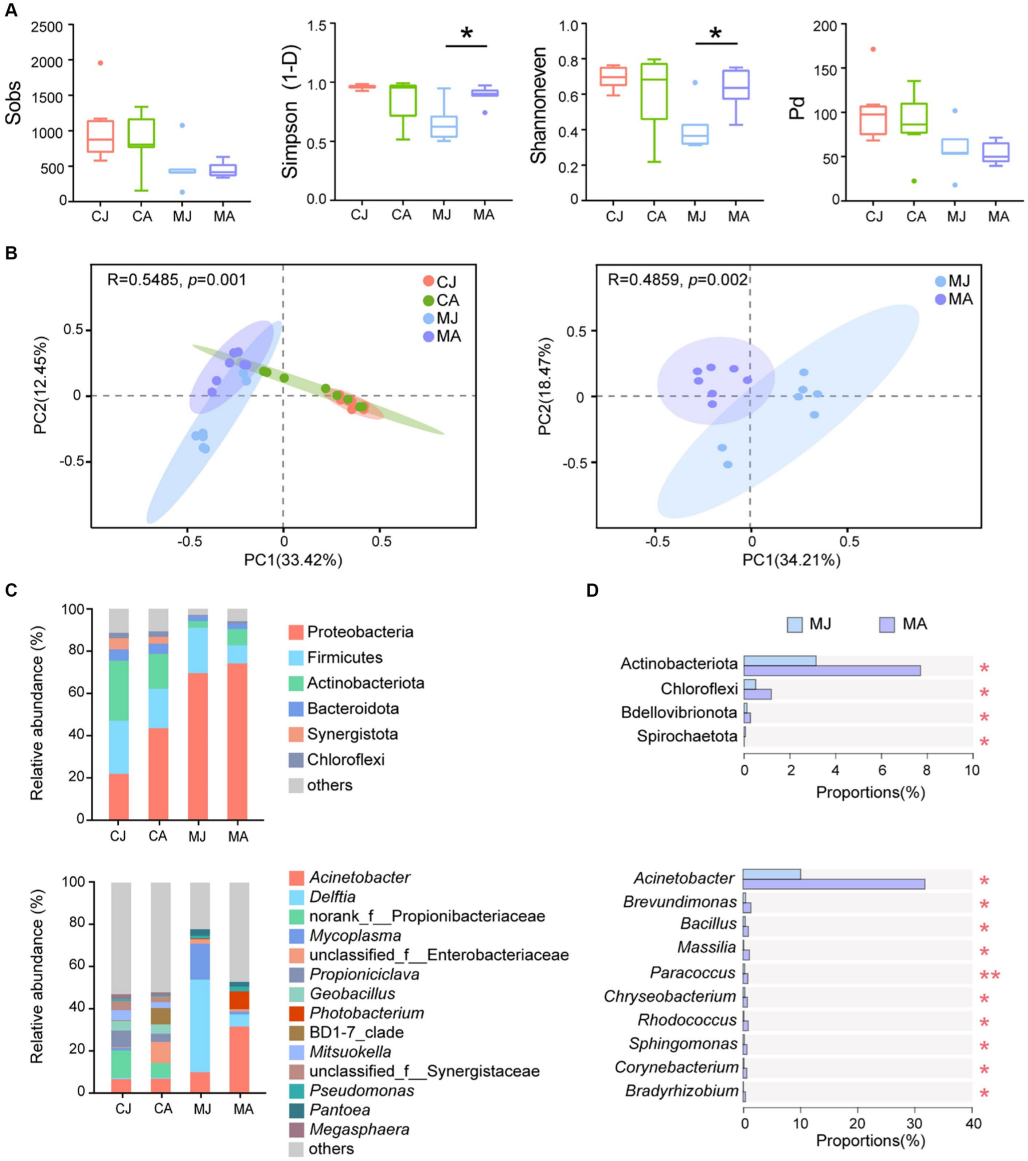
Microbial analysis of gut contents and mucus layer between juvenile and adult tuna. **(A)** Alpha diversity analysis of microbial community. Statistical difference of α-diversity indices was tested by Welch’s t-test. **(B)** PCoA analysis based on Bray–Curtis distance. **(C)** Relative abundance of microbiotas at the phylum and genus level. OTUs with low relative abundance (<2%) were clustered into “others.” **(D)** Wilcoxon rank-sum test bar plot at the phylum and genus level of abundant species. *p ≤ 0.05; **p ≤ 0.01. CJ, gut content microbiotas of juvenile tuna; CA, gut content microbiotas of adult tuna; MJ, mucosal microbiotas of juvenile tuna; MA, mucosal microbiotas of adult tuna.

Alpha diversity indices showed that adult tuna had significantly higher community diversity and evenness of mucosal microbiotas than juvenile tuna (Figure 1A; Simpson: *p* = 0.014; Shannoneven: *p* = 0.040). However, the observed OTUs number (Sobs index) and Pd index showed no significant difference between adult and juvenile groups. Mucosal microbiotas were different among adult and juvenile groups, and different from the gut contents microbial community (Figure 1B). No significant difference was found in the microbial α diversity and β diversity of gut contents in the bacterial community.

For bacterial composition, Proteobacteria, Firmicutes, Actinobacteriota, and Bacteroidota were the most abundant phyla of the gut-content microbial community, totally accounting for 80.72% ~ 83.39%, and Proteobacteria and Firmicutes made up 82.63% ~ 91.03% of mucosal microbiotas community (Figure 1C). The proportion of Proteobacteria was higher in mucosal microbiotas than in gut-content microbiotas (CJ vs. MJ: *p* < 0.01; CA vs. MA: *p* > 0.05; Figure 1C). Actinobacteriota and Chloroflexi were significantly higher in the mucus layer of adult tuna (Figure 1D). At the genus level, the most abundant *Acinetobacter* had a significantly higher abundance in adult tunas, with 31.76% in the adult and 10.05% in the juvenile (Figure 1C). Meanwhile, *Brevundimonas*, *Bacillus*, *Massilia*, *Paracoccus*, *Chryseobacterium*, and *Rhodococcus* were all in higher proportions in the adults than juveniles, among the top 20 abundant genera (Figure 1D; Supplementary Figure S1). With regard to gut-content microbiotas, the abundant phyla showed no significant difference between juveniles and adults (Supplementary Figure S2).

### 3.2 Microbial metabolomics analysis

**Figure 2:**
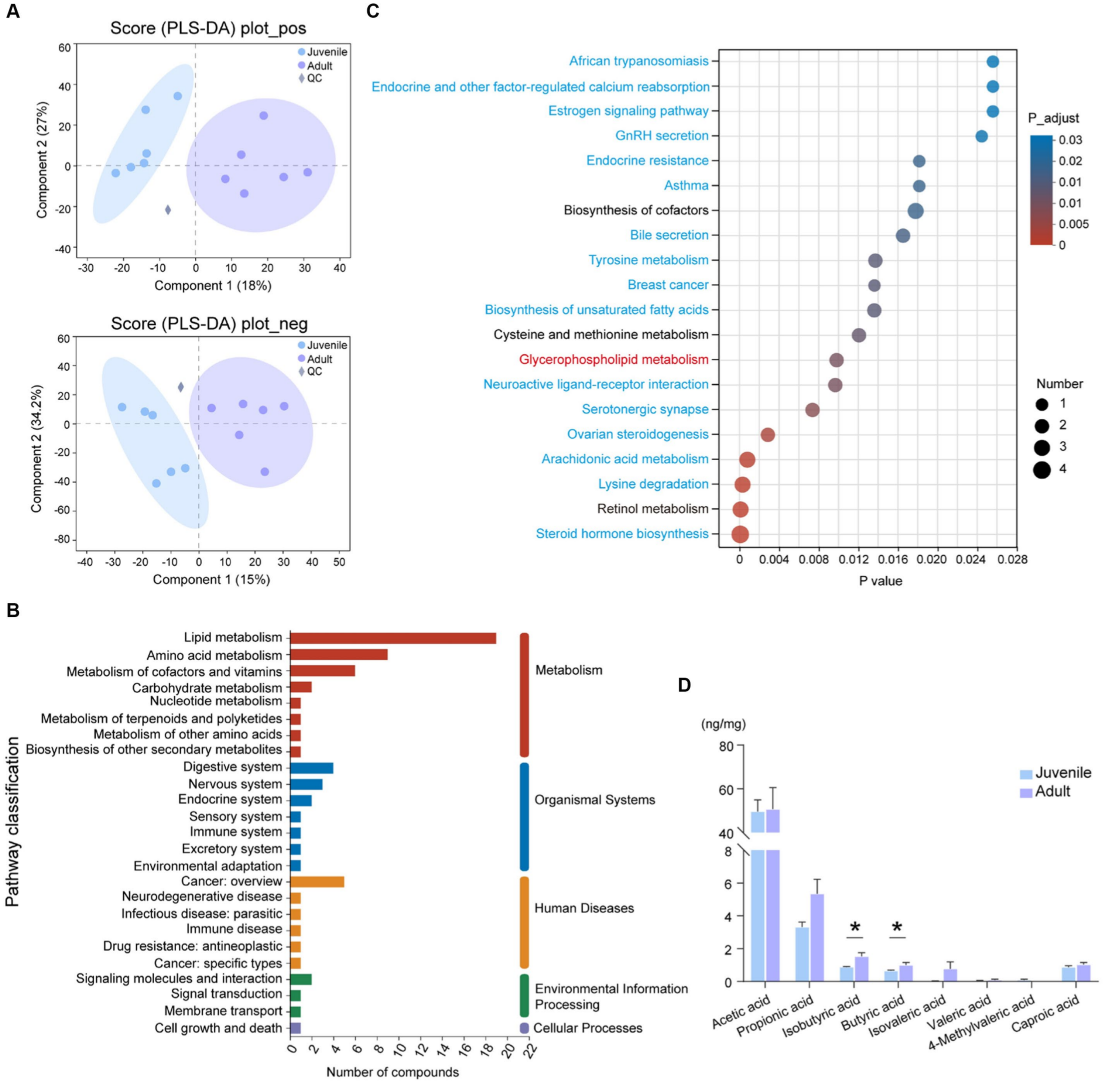
Metabolomic analysis of gut contents between juvenile and adult tunas. **(A)** PLS-DA analysis of samples in positive and negative ion modes. **(B)** KEGG pathway classification of DMs. The vertical axis shows the name of the KEGG pathway at level 2, and the horizontal axis shows the number of DMs annotated in the pathway. The vertical bar at the right with different colors represents different pathway categories. **(C)** Pathway enrichment of DMs by KEGG. The vertical axis shows the name of the KEGG pathway at level 3, and the horizontal axis shows the rich factor. The size and color of the circle show the number and FDR value of the DMs enriched in the pathway. Red pathway was enriched by upregulated DEGs, blue pathways were enriched by downregulated DEGs, and black pathways were enriched by both up- and downregulated DEGs. **(D)** The comparison of the concentration of SCFAs between juvenile and adult tunas. The values were expressed as the means ± SEM. *p < 0.05.

To find the difference in gut microbiota-derived metabolites between juveniles and adults, we carried out the non-targeted LC–MS analysis. Among a total of 2,107 origin metabolites, there were 184 DMs were selected in the positive ion mode and 88 DMs were detected in the negative ion mode (Supplementary Table S3). PLS-DA plot showed a significant separation between juvenile and adult groups (Figure 2A). Compared with the juvenile tuna, the adults had 114 upregulated metabolites and 158 downregulated metabolites (Supplementary Table S3). Among them, 98 DMs were classed into lipids and lipid-like molecules, and 39 DMs were organic acids and derivatives. The top 10 metabolites that contributed most to the difference between the two groups were (8R,9R,10R,13S,14S)-10,13-Dimethyl-2,3,4,7,8,9,11,12,14,15,16,17-dodecahydro 1H-cyclopenta[a]phenanthrene-3,7,17-triol, LysoPA(8,0/0:0), LysoPC(14,0/0:0), (Z)-2-Methyl-2-butene-1,4-diol 4-O-beta-D-Glucopyranoside, L-Cystathionine, Docosapentaenoic acid (22n-3), N-(2-hydroxy-3-methoxy-2-methylpropyl)-2-(2-methoxyphenoxy)acetamide, Alanine lactate pyruvate, 7,10,13,16,19-Docosapentaenoic acid, and Resolvin D1.

A total of 272 DMs were annotated against with KEGG database, the “lipid metabolism” (19 DMs), “amino acid metabolism” (9 DMs), “metabolism of cofactors and vitamins” (6 DMs), and “digestive system” (4 DMs) pathway were the top categories with most clustered DMs (Figure 2B). The identified DMs were further enriched in 45 significant KEGG pathways, such as “steroid hormone biosynthesis,” “retinol metabolism,” “lysine degradation,” “arachidonic acid metabolism,” and “glycerophospholipid metabolism” (Figure 2C).

The targeted SCFA analysis showed that acetic acid had the highest concentration in the gut content of both adult and juvenile tunas. The concentration of butyric acid and isobutyric acid was significantly higher in the adult tunas than in juvenile individuals (Figure 2D).

### 3.3 Intestinal transcriptomics analysis

A total of 324.49 M raw sequencing reads were generated with 54.07 M on average (Supplementary Table S4). After quantity controlling, 314.14 M clean reads with a total of 45.40 Gb clean bases were acquired from tuna gut tissue and Q30 is 94.90%. The average GC content was 49.32%. Transcriptome assembly resulted in a total of 28,269 genes.

**Figure 3:**
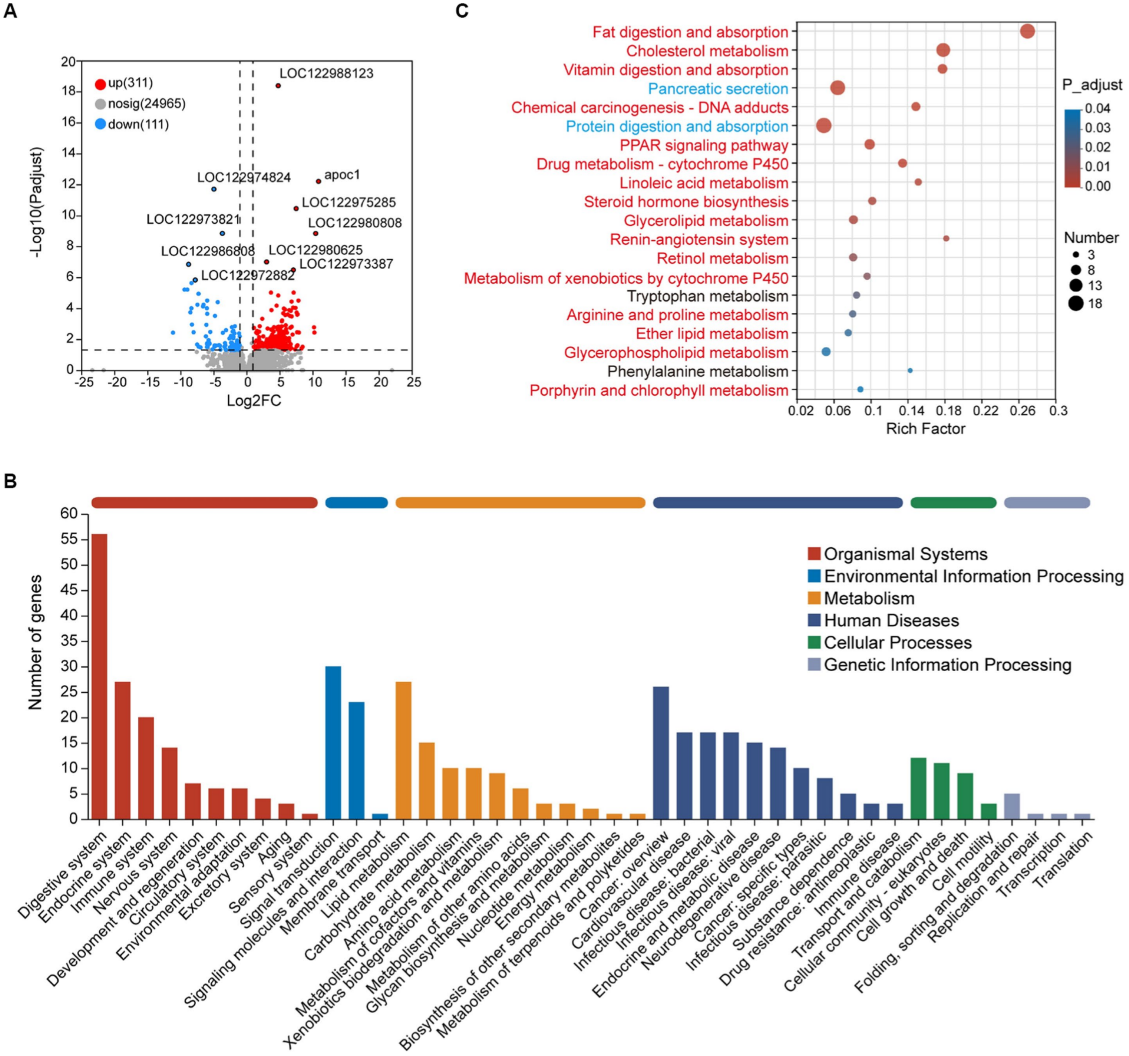
Transcriptome analysis of intestinal DEGs between juvenile and adult tunas. **(A)** Volcano plot of DEGs. **(B)** KEGG pathway classification of DEGs. The vertical axis shows the name of the KEGG pathway at level 2, and the horizontal axis shows the number of the DEGs annotated in the pathway. The horizontal bar with different colors above represents different pathway categories. **(C)** KEGG pathway enrichment of DEGs. The vertical axis shows the name of the KEGG pathway at level 3, and the horizontal axis shows the rich factor. The size of the circle shows the number of the DEGs enriched in the pathway. The color of the circle shows the FDR value. Red pathways were enriched by upregulated DEGs, blue pathways were enriched by downregulated DEGs, and black pathways were enriched by both up-and downregulated DEGs.

GSEA results showed that the genes in the metabolism-associated pathway in the adult group were observed upregulated, such as carbohydrate metabolism and lipid metabolism pathways (Supplementary Table S5; Figure S3). To further evaluate the difference between the two groups, 422 DEGs were obtained for analysis, including 311 upregulated and 111 downregulated DEGs in adult tunas compared with juvenile tuna (Figure 3A). They were mainly classified into five categories of the KEGG metabolic pathway, and the “digestive system” pathway contained the most DEGs (56). Metabolism pathways with more DEGs were “lipid metabolism,” “carbohydrate metabolism,” and “amino acid metabolism” (Figure 3B). The top 20 enriched KEGG pathways of DEGs are shown in Figure 3C. Among them, the upregulated DEGs in adult tunas were mainly involved in lipid metabolism, such as “fat digestion and absorption,” “cholesterol metabolism,” “linoleic acid metabolism,” “steroid hormone biosynthesis,” “glycerolipid metabolism,” “retinol metabolism,” and “glycerophospholipid metabolism.” In the juvenile group, the upregulated DEGs were involved in “protein digestion and absorption” and “pancreatic secretion.” The different nutrition metabolism pathways may also be convinced by digestive enzyme activity results, as tuna’s intestinal lipase had higher enzyme activity in the adult than the juvenile, and trypsin had higher enzyme activity in the juvenile (Supplementary Figure S4).

To verify the expression patterns of DEGs in juveniles and adults, RT-qPCR was run on 14 DEGs detected from transcriptome analysis, including 11 upregulated DEGs (*chka*, *pcyt1*, *pla2*, *plb*, *lpiat*, *apoc1*, *apoa4*, *apoe*, *cd36*, *fabp*, and *dagt*) involved in lipid metabolism, and 3 downregulated DEGs (*prss*, *cela*, and *cpa*) involved in protein digestion and absorption. All the selected genes showed the same expression patterns as that in RNA-Seq, which indicated the reliability of RNA-Seq data (Supplementary Figure S5).

### 3.4 Correlation of differential bacteria, DMs, and DEGs

**Figure 4:**
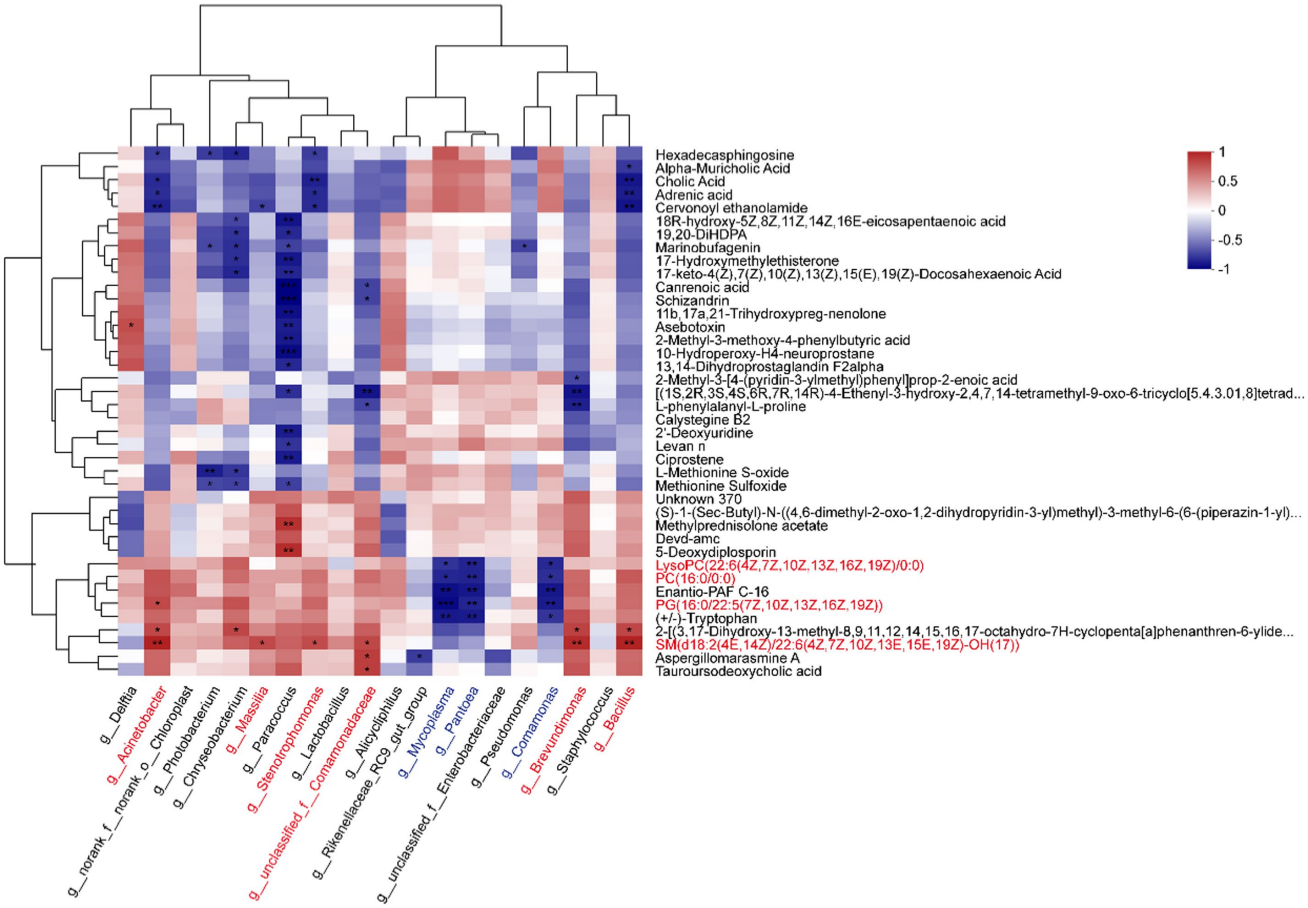
Correlation heatmap between metabolites and microbiotas. The top 20 abundant genera and top 40 abundant DMs were analyzed by the Pearson correlation algorithm (*p < 0.05, **p < 0.01, ***p < 0.001).

According to the Pearson correlation analysis (Figure 4; Supplementary Table S6), the most abundant genera *Acinetobacter*, *Massilia*, *Stenotrophomonas*, *Brevundimonas*, and *Bacillus* showed positive relationships with glycerophospholipid, such as LysoPC (22:6(4Z,7Z,10Z,13Z,16Z,19Z)/0:0), PC (16,0/0,0), and PG (16:0/22:5(7Z,10Z,13Z,16Z,19Z)). However, *Mycoplasma*, *Pantoea*, and *Comamonas* were negatively related to them. Furthermore, sphingomyelin (SM (d18:2(4E,14Z)/22:6(4Z,7Z,10Z,13E,15E,19Z)-OH(17))) was significantly positive-related to *Acinetobacter*, *Massilia*, *Stenotrophomonas*, g__unclassified_f__Comamonadaceae, *Brevundimonas*, and *Bacillus*. *Delftia* was positively related to glycerophospholipid, and negatively related to sphingomyelin.

**Figure 5:**
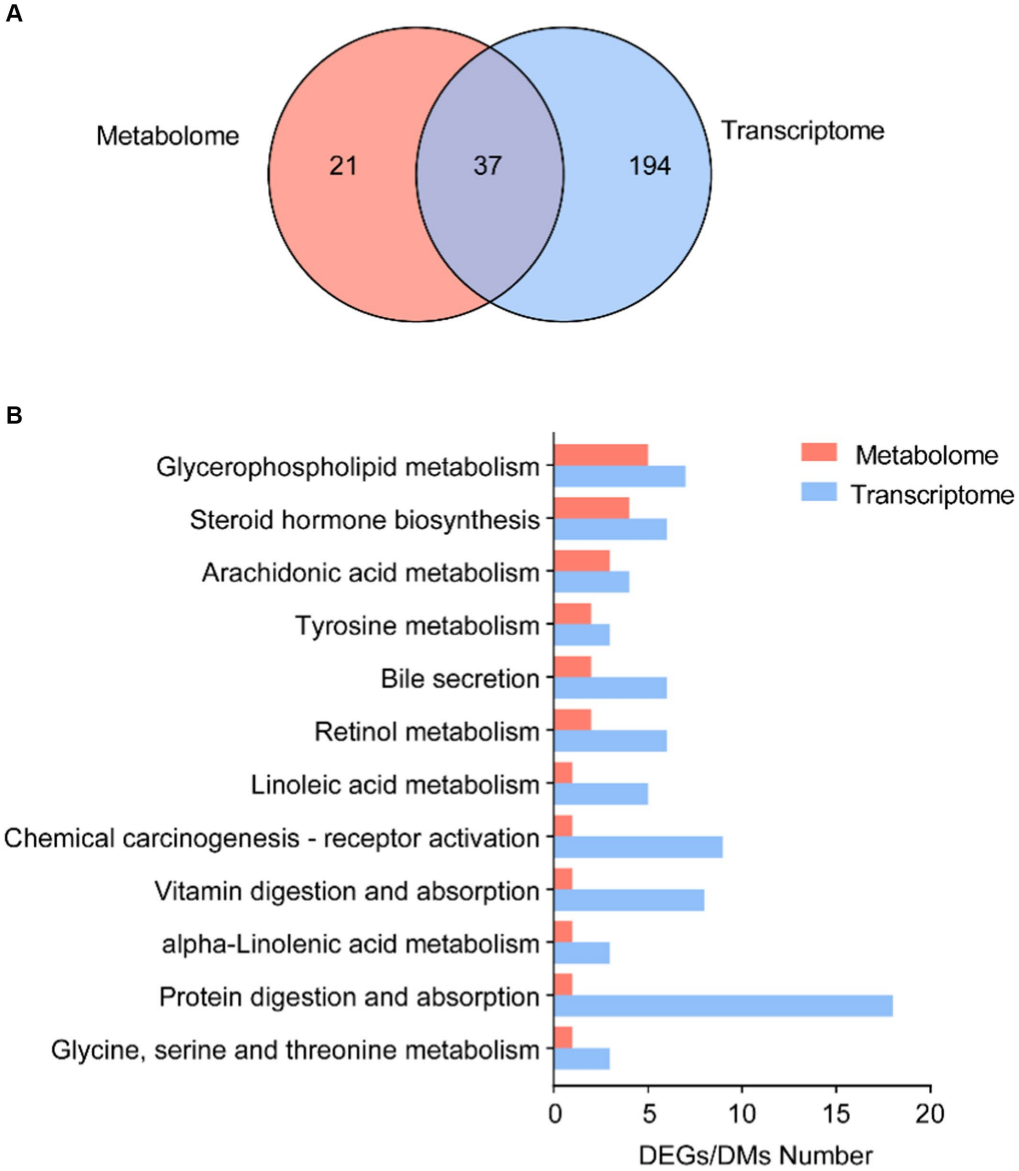
Correlation analysis of microbial DMs and DEGs. **(A)** Number of shared KEGG annotation pathways in metabolome and transcriptome. **(B)** Shared KEGG enrichment pathways in DEGs and DMs.

We further focused on the shared pathways of microbiota-derived metabolites and DEGs in the transcriptome. There were 37 shared pathways annotated in both metabolome and transcriptome (Figure 5A), containing 7 “lipid metabolism” pathways, 3 “amino acid metabolism” pathways, and 3 “digestive system” pathways (Supplementary Table S7). In addition, both of them significantly enriched in 12 common KEGG pathways, such as “glycerophospholipid metabolism,” “steroid hormone biosynthesis,” “arachidonic acid metabolism,” “bile secretion,” “linoleic acid metabolism,” and “alpha-linoleic acid metabolism” (Figure 5B).

**Figure 6:**
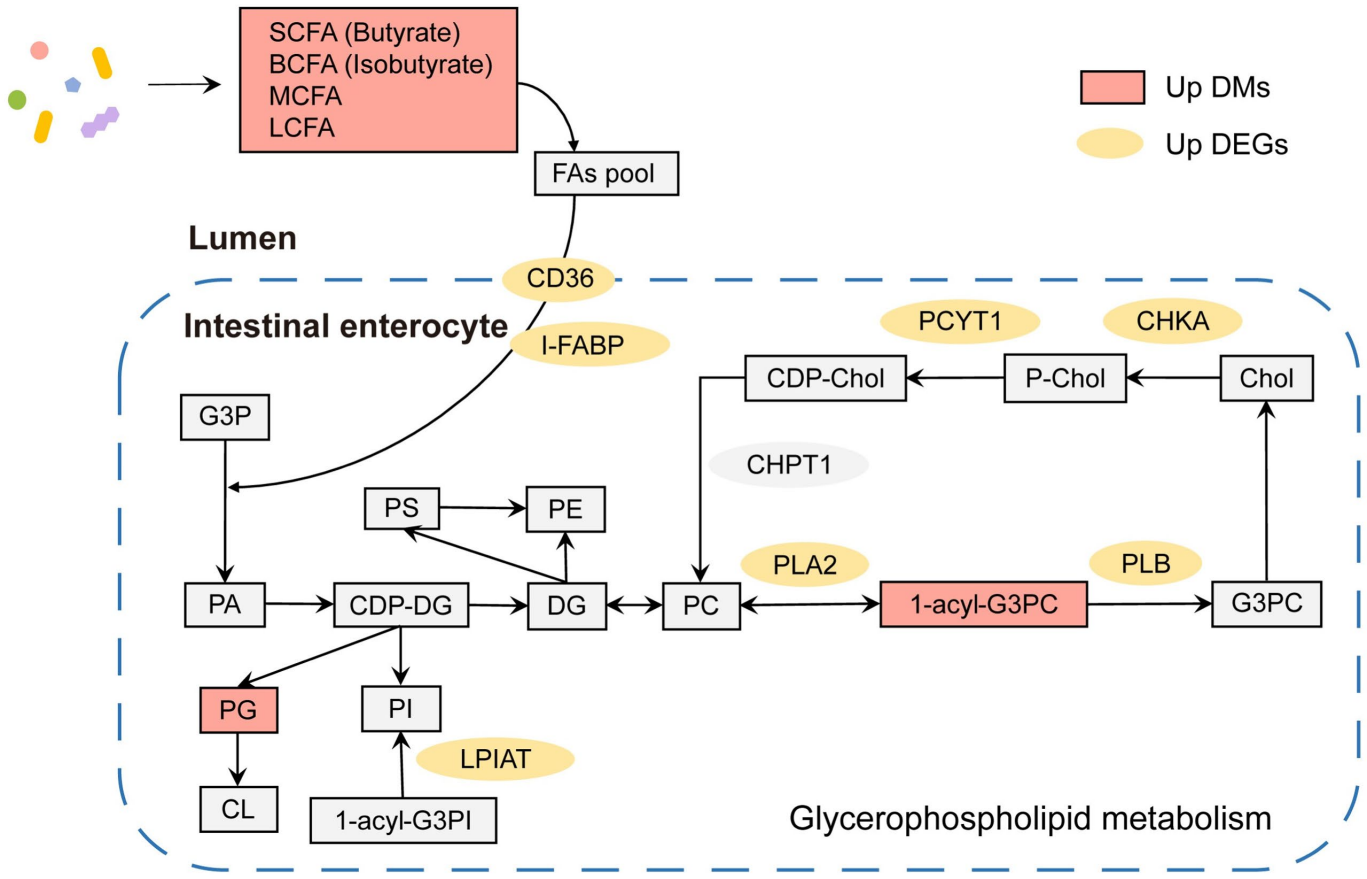
Glycerophospholipid metabolism pathway in adult tuna.

In summary, both microbial metabolites and gene expression in hosts showed significant differences in lipid metabolism between adult and juvenile tuna. As shown in Figure 6, in adult, there were several key lipid molecules involved in glycerophospholipid metabolism, including LysoPC (17,0/0:0), LysoPC (14,0/0:0), PC (16,0/0,0), LysoPC (22:6(4Z,7Z,10Z,13Z,16Z,19Z)/0:0), and PG (16:0/22:5(7Z,10Z,13Z,16Z,19Z)). In the intestinal lumen, fatty acids (such as SCFA, BCFA, MCFA, and LCFA) generated by gut bacteria provided a fatty acyl group and transported into intestinal enterocytes with the help of fatty acid translocase (FAT/CD36) and fatty acid-binding protein (FABP) to participate in the process of synthesis phosphatidic acid (PA) by 3-phosphoglyceraldehyde (G3P). PA can be a premise for the next biosynthesis of kinds of phosphoglycerides, such as phosphatidylglycerol (PG). In addition, the upregulation of 1-acyl-sn-glycero-3-phosphocholine (1-acyl-G3PC) and enzymes (PLA2, PLB, CHKA, and PCYT1) of adult tunas promoted the cycling and biosynthesis of phosphatidylcholine (PC). In addition, the upregulated LPIAT could stimulate the biosynthesis of phosphatidylinositol (PI).

## 4 Discussion

A better understanding of the function of host-associated microbiotas and the relationship between microbiotas and host is essential for physiological research and aquaculture of tuna. In this study, we revealed that the development stage was an important variable factor shaping the gut microbial composition and diversity, and thus the metabolomic and transcriptomic expression profiles were different between adults and juveniles.

As an important organ of dietary digestion and absorption, the intestine is considered a complicated micro-ecosystem with vast and diverse microbiotas (Gilbert et al., 2018; Butt and Volkoff, 2019). It has been recognized that the intestinal microbiota is directly involved in the metabolism of carbohydrates, lipids, and proteins, as well as the synthesis of vitamin and secondary bile acids (Brestoff and Artis, 2013; Frampton et al., 2020). For tuna, a set of specialized gut bacteria may evolve specific interactions with the host. *Acinetobacter*, for instance, is one of the most abundant gut bacteria of tunas (Minich et al., 2020; Zou et al., 2023), and it was significantly different in the proportion of relative abundance between adult and juvenile tunas (Figure 1). This study detected a different gut mucosal microbial structure between juveniles and adults, and similar size-specific taxa differences were found in both yellowfin tuna and bigeye tuna, as described in Gadoin et al. (2021). Microbiotas in the mucosal layer are appointed to represent resident bacteria (Kashinskaya et al., 2017), and they showed more microbial diversity in adults than in juveniles. However, no difference was found in gut content microbial diversity and composition between juvenile and adult tunas, which was similar to our previous study in Zou et al. (2023). It is recognized that host-associated factors, such as diet, age, and species, are important factors influencing gut microbial diversity and composition (Ou et al., 2021). As an oceanic top predator, yellowfin tuna has been evidenced to show a two-stanza growth pattern (Dortel et al., 2020), with an increasing growth rate observed in 60 ~ 75 cm fork length (FL) compared to a decreasing trend in 40 ~ 60 cm FL (Eveson et al., 2015). The dietary change may be in favor of the change of nutritional needs depending on the growth stage of the tunas. For example, as Graham et al. (2007) reported, the diet shift of yellowfin tuna in Hawaii happened at 45 ~ 50 cm FL, and less consumption of stomatopoda and more teleosts and mollusca. Thus, the different microbial composition and abundance between the two groups are related to the host dietary shift. In addition, with body size increasing, the major prey components of large-size yellowfin tunas (75 ~ 100 cm FL) have a more similar proportion than small tunas (Graham et al., 2007), which contributed to the community evenness and homeostasis of gut microbiota of adults.

Proteobacteria and Firmicutes are the common phyla in the gut mucus, making up a large part of the gut microbial community (Wang et al., 2018). The two main phyla remained relatively stable in abundance between juveniles and adults, while the abundance of Actinobacteriota showed a significantly higher in the adult tunas, indicating a host-selective role in bacteria enrichment. In our study, different metabolites were regulated by distinct gut members. In detail, the data on intestinal taxonomic compositions revealed that both *Acinetobacter* and *Bacillus* showed a positive correlation with glycerophospholipids and sphingomyelin from Pearson correlation analysis (Figure 4), suggesting that those species in the adult tuna may participate in lipid metabolism. *Bacillus* spp. are commonly used as a probiotic for their remarkably diverse functions in fish health (Kuebutornye et al., 2019). As reported in the previous study of Jang et al. (2023), a *Bacillus* strain isolated from the red sea bream intestine was critical for maintaining intestinal symbiosis and homeostasis and positively altered digestive enzyme activity. In contrast, potential pathogens, *Mycoplasma* and *Comamonas*, were significantly negatively related to the glycerophospholipid and sphingomyelin molecular. It is likely that microbiota-derived metabolites may interact with other gut bacteria, and these bacteria metabolic products may influence the microbial structure (Liu et al., 2022).

The influence of gut bacteria on the host is mainly driven by microbial metabolism, specifically the amino acid, lipid, and carbohydrate metabolic pathways (Gilbert et al., 2018; Butt and Volkoff, 2019). Metabolomic PLS-DA analysis displayed a complete separation of metabolites between the juvenile and adult groups (Figure 2). The elevated levels of lipid and lipid-like molecules suggested that the dominant bacteria may correlate with the improvement of intestinal physiology in adult tuna. In our study, a significantly high level of glyceryl phosphatide and sphingolipids was observed from the top 30 high VIP DMs. One of the glycerophospholipids, PG, is an important component of biological membranes (Dugail et al., 2017), and was upregulated in adult tunas. Although PGs are enriched in the membrane of gut bacteria, we hypothesized that bacterial PG would contribute equally to the juvenile and adult. Sphingolipids are also important components of the biofilm (Lordan and Blesso, 2023). Several sphingolipids, such as sphingomyelin (SM), are considered to be involved in a variety of cellular processes including differentiation, cellular senescence, apoptosis, and proliferation (Ohanian and Ohanian, 2001). Brown et al. (2019) also reported that Bacteroides-derived sphingolipids are negatively related to inflammatory disease and critical for maintaining intestinal homeostasis. Thus, gut microbiotas and their derived metabolites synergistically support intestinal health (Liu et al., 2022).

SCFAs rely heavily on the metabolism of gut microbiotas (Postler and Ghosh, 2017). The gut microbiotas can ferment carbohydrates and proteins of dietary, which contributes to the total SCFA pool, including acetate, propionate, butyrate, and branched-chain fatty acids (BCFAs; Krautkramer et al., 2021). Butyrate can not only as a primary energy source of colonocytes impacting the energy metabolism of the host but also as an epigenetic regulator of gene expression by inhibiting histone deacetylase (Donohoe et al., 2011; Bedford and Gong, 2018; Frampton et al., 2020). In aquaculture practice, butyrate is used as a nutrition element to improve the growth performance and intestinal immunity of fish (Li et al., 2024). In the carnivorous host, producing-BCFAs members are considered to have a vital role in diet amino acids metabolism, promoting the composition of isobutyrate, isovalerate, and isocaproate (Xu et al., 2020). In the present study, the increased butyrate and isobutyrate in adult tunas implied that enriched species may participated in SCFAs production, promoting host metabolism homeostasis and health. Transcriptome analysis provides another perspective on the gene expression of the intestine in adult yellowfin tunas. In this study, the pathways related to lipid metabolism in the adult group were significantly upregulated, such as fat digestion and absorption, cholesterol metabolism, linoleic acid metabolism, steroid hormone biosynthesis, glycerophospholipid metabolism, and peroxisome proliferator-activated receptors (PPAR) signaling pathway (Figure 3C), which also played an important role in regulating lipid metabolism in mature adipocytes by increasing fatty acid trapping (Yu et al., 2021). In adult tunas, *cd36* and *fabp2* were high-expressed in the fat digestion and absorption pathway, which encodes fatty acid translocase and fatty acid-binding protein, respectively, promoting lipid transport into enterocytes (Chen et al., 2001). Apolipoprotein genes, *apoc1*, *apoa4*, and *apoe*, were upregulated, helping fat digestion and absorption and cholesterol metabolism for adults (Huang and Mahley, 2014). Diacylglycerol acyltransferase (DGAT) participated in the catalyzing synthesis of triglyceride (Bhatt-Wessel et al., 2018), which may be beneficial to lipid utilization and energy supply for adults. However, the upregulated DEGs in the juvenile group were involved in protein digestion and absorption, and pancreatic secretion. *Prss* and *cela2* encode trypsinase and elastase respectively, they are members of serine proteases and involved in intestinal protein digestion. Moreover, the intestinal lipase activity of adult tuna was higher than juveniles, but trypsin had an opposite trend (Supplementary Figure S4). It seems that juveniles were more prone to consume protein to harvest energy from dietary, and adults prefer lipids. This may be beneficial to provide essential fatty acids for the gonad development of adult tuna. As a previous study reported, adding arachidonic acid, eicosapentaenoic acid, and docosahexaenoic acid could improve the quality of eggs and offspring of European eel (Kottmann et al., 2020) and Atlantic cod (Norberg et al., 2017). Apart for beneficial in development, lipids can provide the main fuel for aerobic exercise in fish swimming (Kieffer et al., 1998), which is adapted to the long-distance swimming habit of large-size tunas.

Conjoint analysis with metabolome, the enriched pathways of both differential expressed genes and metabolites were remarkably related to the regulation of glycerophospholipids metabolism (Figure 5B). It was an important pathway in maintaining the stability and integrity of membranes, brain development, and regulating lipid metabolism (Zhang et al., 2019). The phospholipases in cells might be involved in the modulations of glycerophospholipids. In adult tunas, phospholipases, PLA2 and PLB, involved in the glycerophospholipids metabolism were upregulated to promote the synthesis of choline into PC (Figure 6). Choline kinase alpha (CHKA) and choline-phosphate cytidylyltransferase (PCYT) are two enzyme-catalyzed synthesis PC (Figure 6). PLA2 and PLB are crucial for cellular responses, including phospholipid digestion and metabolism, host defense, and signal transduction (Murakami et al., 2017). With the contribution of gut microbiotas, the fatty acids pool in the intestinal lumen was enriched and went into enterocytes by simple diffusion, promoting the synthesis of phosphatidic acid (PA), which is a premise for the next step of the biosynthesis of many different glycerophospholipids (Huang and Freter, 2015). For further study, *in vitro* and *in vivo* experiments should be conducted to validate the relationship between metabolites and genes, including the gene knockdown/over-expression and physiological indicators.

## 5 Conclusion

Our results demonstrated that adult tunas have higher gut microbial diversity and better lipid metabolism ability than juveniles. The preference for nutrient utilization between adult and juvenile tuna is changed with the ontogenetic stage. In the adult tunas, the elevated levels of lipid-like molecules provided potential functional metabolic benefits to the adults, which may be necessary to improve the host physiology, such as adult gonad development. Our findings highlighted the important roles of gut microbiota and the complex interactions of different gut members that contribute to endothermic and high-speed swimming marine fish species.

## Data availability statement

The datasets presented in this study can be found in online repositories. The names of the repository/repositories and accession number(s) can be found at: https://www.ncbi.nlm.nih.gov/, BioProject ID: PRJNA1018001; https://www.ncbi.nlm.nih.gov/, BioProject ID: PRJNA1017392.

## Ethics statement

Ethical approval was not required for the study involving animals in accordance with the local legislation and institutional requirements because Ethical review and approval were not required for the animal study because all tunas were collected by commercial fishing.

## Author contributions

YiZ: Data curation, Investigation, Methodology, Validation, Writing – original draft. YaZ: Visualization, Writing – review & editing. DW: Visualization, Writing – review & editing. ZL: Investigation, Funding acquisition, Writing – review & editing. JX: Investigation, Writing – review & editing. HH: Writing – review & editing. QF: Writing – review & editing. ZG: Funding acquisition, Writing – review & editing.

## Footnotes

^1^ http://www.hmdb.ca/

^2^ https://metlin.scripps.edu/

^3^ https://github.com/jstjohn/SeqPrep

^4^ https://github.com/najoshi/sickle

^5^ http://ccb.jhu.edu/software/hisat2/index.shtml

^6^ https://ccb.jhu.edu/software/stringtie/index.shtml?t=example

^7^ http://www.broad.mit.edu/GSEA
