# Supplementary material for: Multi-omics analysis revealed the differences in lipid metabolism of the gut between adult and juvenile yellowfin tuna (Thunnus albacares)
Source: Front Microbiol. 2024 Jan 11;14:1326247. doi: 10.3389/fmicb.2023.1326247 (PMC10808786; doi:10.3389/fmicb.2023.1326247)
Supplement: Supplementary file 1 [file Data_Sheet_1.docx]

**Supplementary Figures**


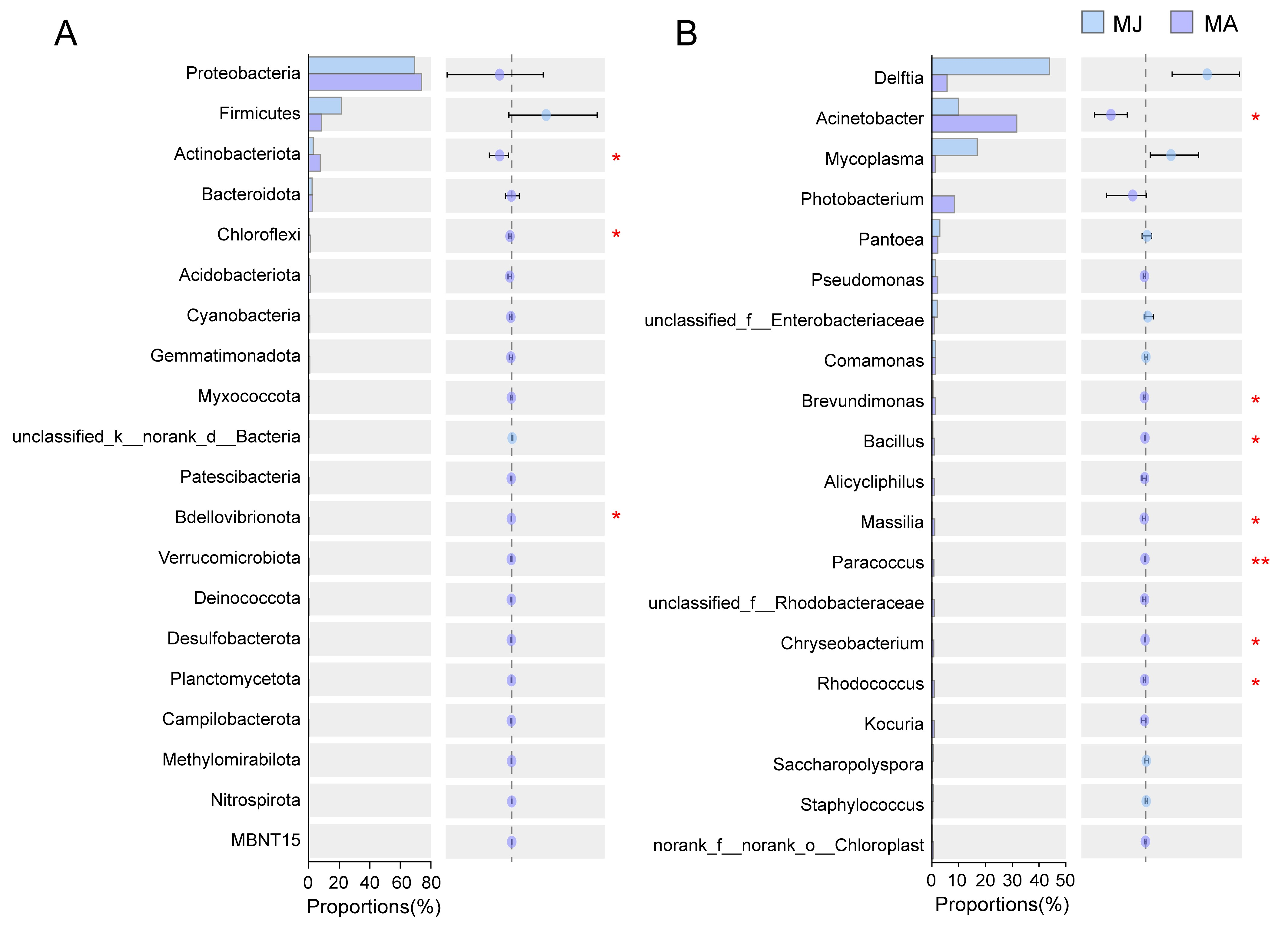


**Figure S1**. Difference of top 20 abundant microbiotas of gut mucus at the (A) phylum level and (B) genus level between the two groups. Statistical difference was test by Wilcoxon rank-sum test. MJ: gut mucosal microbiotas of juvenile tuna; MA: gut mucosal microbiotas of adult tuna. * *p* < 0.05, ** *p* < 0.01.


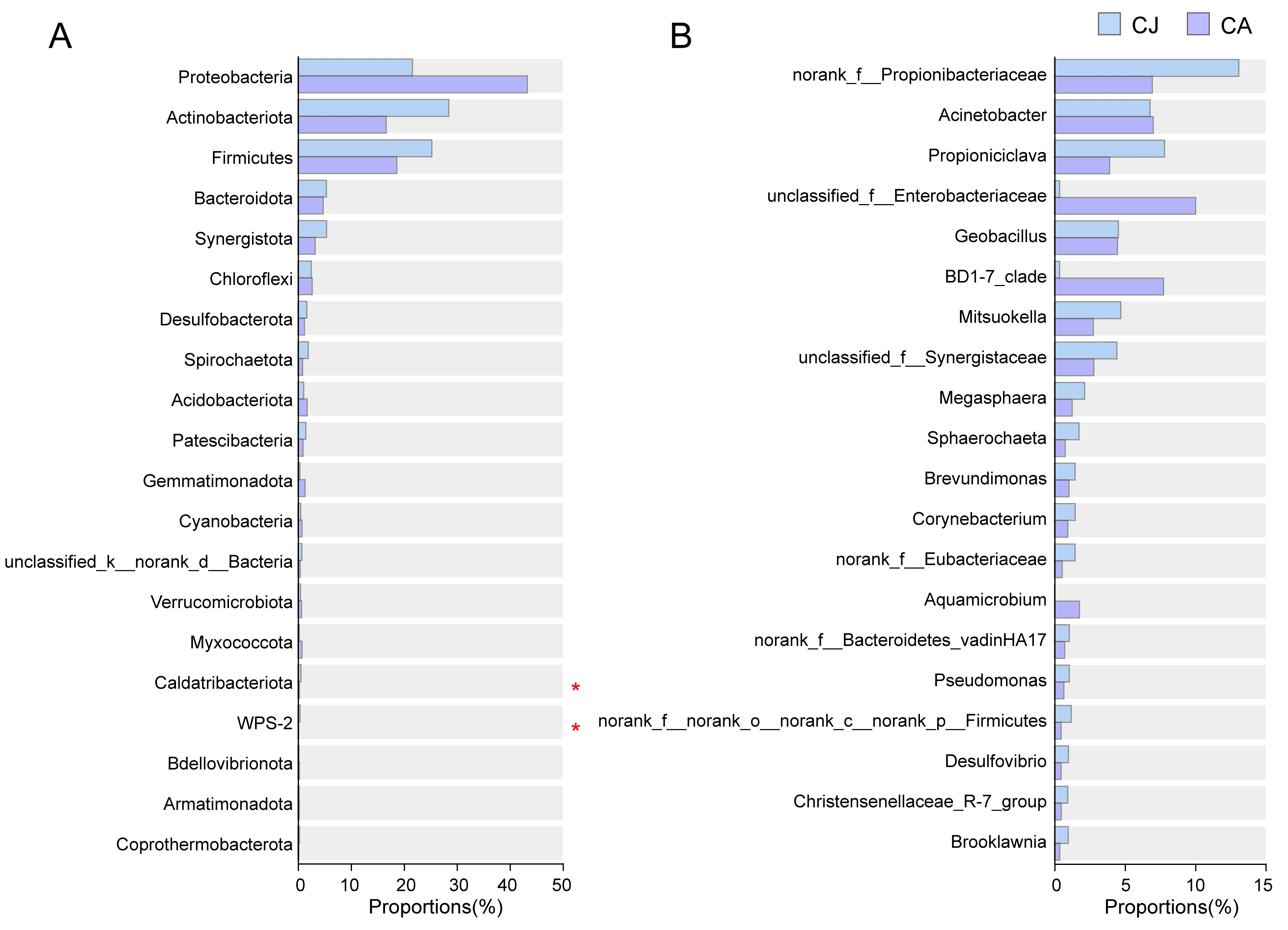


**Figure S2**. Difference of top 20 abundant microbiotas of gut contents at the (A) phylum level and (B) genus level between the two groups. CJ: gut content microbiotas of juvenile tuna; CA: gut content microbiotas of adult tuna. Statistical difference was test by Wilcoxon rank-sum test. * *p* < 0.05, ** *p* < 0.01.


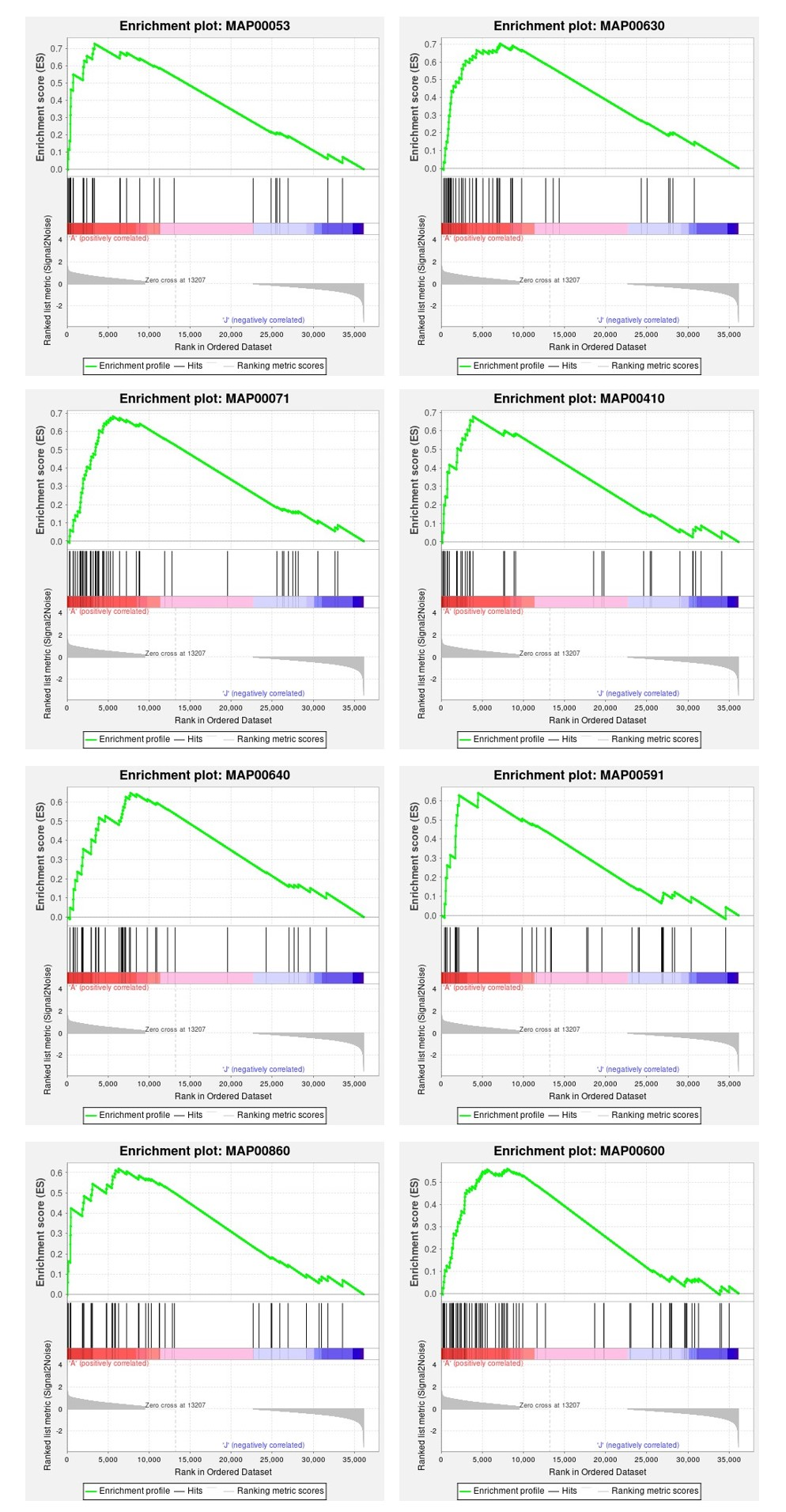


**Figure S3**. GSEA enrichment plot of metabolism-associated pathway.


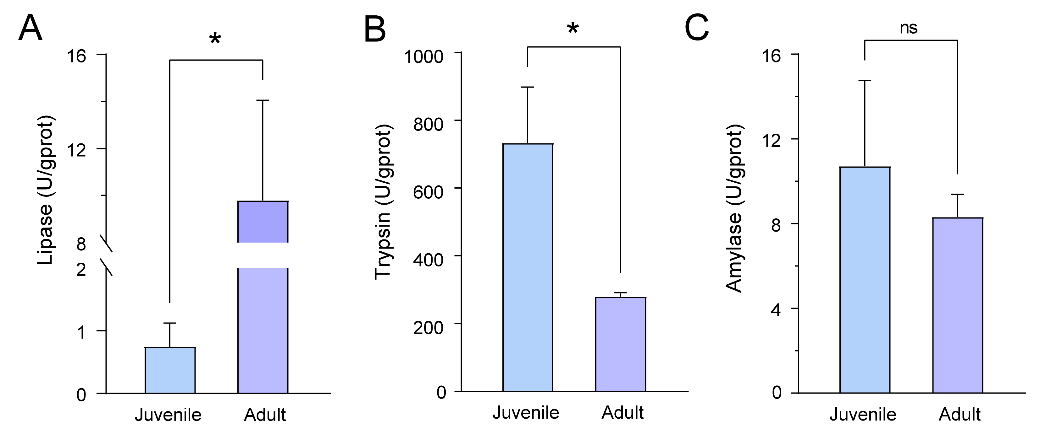


**Figure S4**. Intestinal digestive enzymes activities of juvenile and adult tunas. (A) lipase enzymes activity, (B) trypsin enzymes activity, (C) α-amylase enzymes activity. * *p* < 0.05.


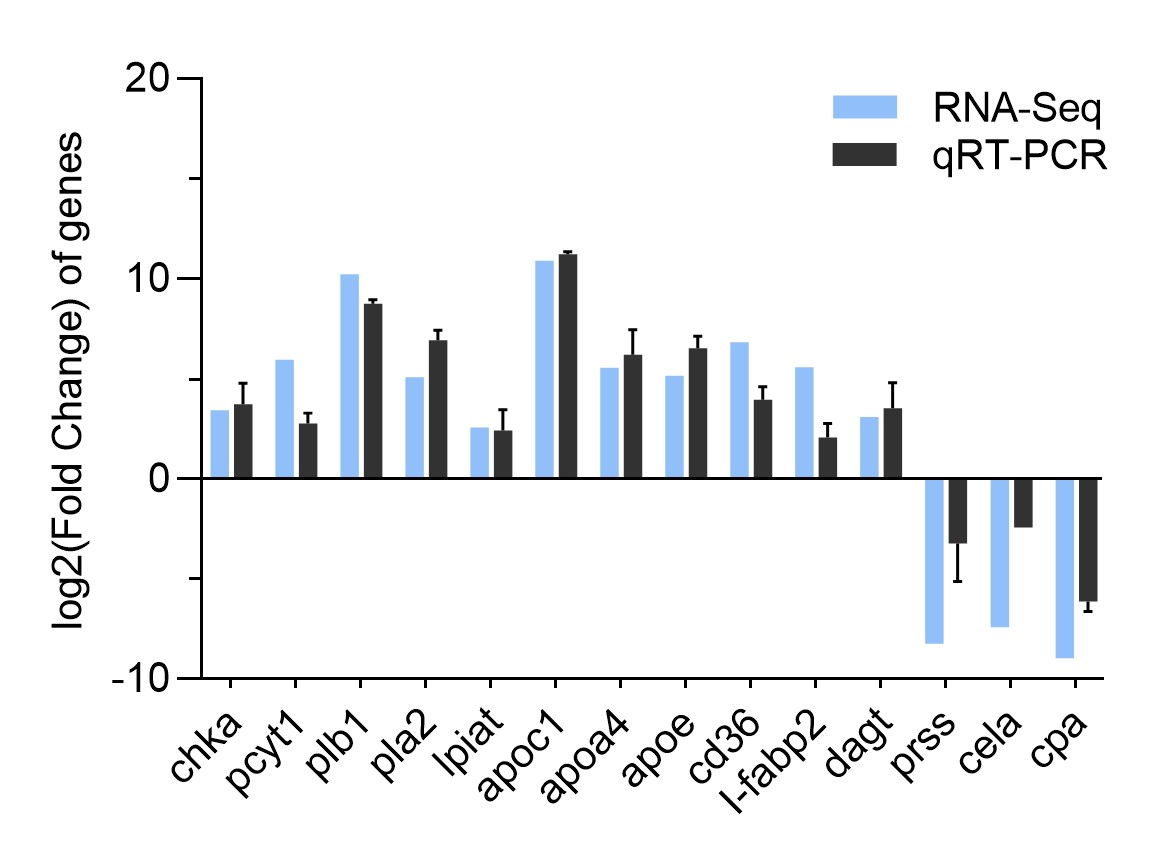


**Figure S5**. Verification of key DEGs.
